# Correction: Improved isoprene detection performance of Si-doped WO_3_ films deposited by sputtering and post-annealing

**DOI:** 10.1039/d4ra90131b

**Published:** 2024-11-07

**Authors:** Pin-Kuan Lin, Yi Qin, Xiaoding Qi, Liji Huang

**Affiliations:** a Department of Materials Science and Engineering, National Cheng Kung University Tainan City 70101 Taiwan xqi045@ncku.edu.tw; b Centre for Micro/Nano Science and Technology, National Cheng Kung University Tainan City 70101 Taiwan; c Siargo Ltd Santa Clara California 95054 USA

## Abstract

Correction for ‘Improved isoprene detection performance of Si-doped WO_3_ films deposited by sputtering and post-annealing’ by Pin-Kuan Lin *et al.*, *RSC Adv.*, 2024, **14**, 13618–13627, https://doi.org/10.1039/D4RA00184B.

The authors regret an error in Fig. 1 where Fig. 1b was mistakenly replaced by another image used as a scaling reference.

The corrected Fig. 1, with the original data for Fig. 1b, is shown below.

**Fig. 1:**
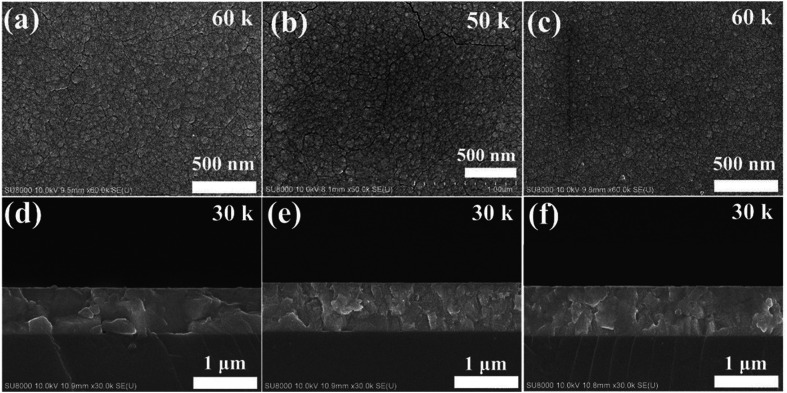
Surface SEM images for (a) 0SiW, (b) 6SiW and (c) 11SiW. Cross-sectional SEM images for (d) 0SiW, (e) 6SiW and (f) 11SiW.

An independent expert has viewed the new data and has concluded that it is consistent with the discussions and conclusions presented.

The Royal Society of Chemistry apologises for these errors and any consequent inconvenience to authors and readers.

